# Postmenopausal ovarian hyperandrogenism of surgically treated patients: a case report and scoping review with individual patient’s data analysis

**DOI:** 10.3389/fendo.2025.1495930

**Published:** 2025-08-01

**Authors:** Angelo Forte, Lanfranco D’Elia, Carmine De Luca, Antonella Fiore, Antonio Barbato, Veronica Abate, Anita Vergatti, Nunzia Verde, Gianpaolo De Filippo, Pietro Venetucci, Maria Chiara De Angelis, Rosa Maria Di Crescenzo, Francesca Grasso, Perruolo Giuseppe, Pietro Formisano, Attilio Di Spiezio Sardo, Rosario Pivonello, Domenico Rendina

**Affiliations:** ^1^ Department of Clinical Medicine and Surgery, Federico II University, Napoli, Italy; ^2^ Service d’Endocrinologie et Diabétologie Pédiatrique, Assistance Publique-Hôpitaux de Paris, Hôpital Robert-Debré, Paris, France; ^3^ Advanced Biomedical Sciences Department, University Federico II of Naples, Naples, Italy; ^4^ Department of Neuroscience, Reproductive Sciences and Dentistry, Federico II University, Naples, Italy; ^5^ Department of Advanced Biomedical Sciences, Federico II University, Naples, Italy; ^6^ Department of Translational Medical Sciences, Federico II University of Naples, Naples, Italy; ^7^ Department of Public Health, Federico II University, Naples, Italy

**Keywords:** androgens, testosterone, dehydroepiandrosterone sulphate, 17-OH progesterone, follicle-stimulating hormone, luteinizing hormone

## Abstract

**Introduction:**

Postmenopausal hyperandrogenism (PH) is a rare clinical condition caused by relative or absolute androgen excess after menopause. Tumorous or non-tumorous ovarian diseases can cause PH.

**Methods:**

In this two-section hybrid study, the first section describes the case of a patient with PH caused by an ovarian disease and surgically treated. The second section shows the results of a scoping review with individual patient data (IPD) analysis, which was performed to define the biochemical and clinical features of PH patients with tumorous or non-tumorous ovarian diseases surgically treated. All PH cases caused by anything but ovarian disease and/or without surgical indication and/or without histological diagnosis were excluded.

**Results:**

Due to imaging suspicion, our PH patient underwent robotic hysterectomy with bilateral ovariectomy. A Leydig cell tumor stage 1A was diagnosed. At 6 months after surgery, the PH was resolved. Overall, the IPD analysis included 280 PH patients with ovarian diseases (oPH) surgically treated. Among them, histological examination showed 174 tumorous oPH and 106 non-tumorous oPH. Patients with tumorous oPH showed lower body mass index and lower levels of luteinizing hormone (LH) and follicle-stimulating hormone (FSH), as well as higher levels of testosterone, dehydroepiandrosterone sulfate (DHEA-S), 17-OH progesterone, and estradiol compared with non-tumorous oPH patients. We defined the levels of testosterone (≥9.8 nmol/L), LH (≤15 mUI/ml), FSH (≤35 mUI/ml), and DHEA-S (≥1.6 μmol/L) able to differentiate between tumorous and non-tumorous oPH patients with suitable sensitivity (≥68.6%) and specificity (≥72.7%). No PH recurrence was described after surgery.

**Discussion:**

The study results provide useful biochemical parameters to support the diagnosis of ovarian tumor in patients with oPH.

## 1 Introduction

Postmenopausal hyperandrogenism (PH) is a rare clinical condition caused by relative or absolute androgen serum excess after menopause from an adrenal or an ovarian source (1). It is further amplified by a decrease in the sex hormone-binding globulin (SHBG) levels, which increases the free androgen index (2). PH clinically appears with symptoms and signs of virilization. According to the criteria proposed by Markopoulos and colleagues, there are several causes of PH so that it can be generally categorized into non-tumorous or tumorous (3). Non-tumorous PH includes polycystic ovary syndrome, congenital adrenal hyperplasia, ovarian hyperthecosis, states of insulin resistance including obesity, endocrinopathies such as Cushing’s syndrome and acromegaly, and, finally, the use of certain drugs such as testosterone and its analogues, valproic acid, and oxcarbazepine. On the other hand, tumorous PH includes adrenal or ovarian neoplasms (3). As PH can be the sole manifestation of several conditions and due to the lack of standardized diagnostic procedures, a two-section hybrid study was performed. In the first section, we report on the case of a patient with PH caused by an ovarian disease and who was surgically treated. In the second section, a scoping review with individual patient data (IPD) analysis collected and analyzed all PH cases of ovarian source (oPH) requiring surgery and with histological diagnosis to better define the clinical characteristics of the patients with oPH described so far. To our best knowledge, these data are not available in the international literature yet.

## 2 Materials and methods

### 2.1 Case report

Considering that some clinical characteristics and parameters, such as body mass index (BMI), ethnicity (4, 5), and homeostatic model assessment of insulin resistance (HOMA-IR), can influence the serum levels of sexual and pituitary hormones, we enrolled a control group (controls) according to the following criteria. Controls were chosen from among postmenopausal women consecutively referred to the Department of Clinical Medicine and Surgery of Federico II University, from January 1, 2024, to March 31, 2024. The inclusion criteria for the controls were: age ranging from 55 to 65 years, BMI ranging from 28.0 to 32.0 kg/m^2^, and personal history of type 2 diabetes mellitus (T2DM). All participants provided informed written consent for participation in the study. The serum levels of estradiol (E), follicle-stimulating hormone (FSH), luteinizing hormone (LH), testosterone (T), androstenedione (A), and dehydroepiandrosterone sulfate (DHEA-S) were measured with the Advia Centaur XT Immunoassay System Analyzer (Siemens, Munich, Germany), which uses competitive (for E) or direct (for T, A, DHEA-S, FSH, and LH) immunoassay. The chemiluminescent acridinium ester technology was used for reaction quantification. SHBG and 17-OH progesterone (17-OHP) were measured using the IDS iSYS analyzer, which uses direct immunoassay. The chemiluminescent acridinium ester technology was used for reaction quantification. The serum concentration of inhibin B (Inb) was measured using competitive chemiluminescence immunoassay with the YLO IFlash analyzer. The intra- and inter-assay coefficients of variation were 2.4% and 1.4% for FSH, 2.7% and 2.3% for LH, 4.3% and 6.6% for T, 3.0% and 4.6% for SHBG, 3.2% and 4.3% for A, 5.5% and 6.9% for DHEA-S, 4.2% and 1.9% for E, 2.4% and 6.2% for 17-OHP, 5.1% and 4.6% for Inb, respectively.

### 2.2 Scoping review with IPD analysis

#### 2.2.1 Data sources and search strategy

The scoping review was planned, conducted, and reported according to the Preferred Reporting Items for Systematic reviews and Meta-Analyses (PRISMA) statement (6). A scoping search of the medical literature was performed in Medline, Google Scholar, Google Book, and Cochrane Library (last conducted search February 25, 2024) using the following term: “Postmenopausal Hyperandrogenism.” There were no language restrictions. The reference lists of all identified articles were searched for further relevant publications.

#### 2.2.2 Study selection

Eligible studies were case reports, cases series, and review articles. The review articles were selected for this study to evaluate the state-of-the-art and to find additional articles that could not be found initially by checking the references. Because some individual case reports were included as part of a subsequent case series by the same authors, duplicates were excluded. The predetermined inclusion criteria were: patients with oPH requiring surgery due to severe hyperandrogenism or because of ovarian imaging suspicion. The exclusion criteria were: women with known cause of ovarian hyperandrogenism not subject to surgical treatment, for instance polycystic ovary syndrome, premenopausal hyperandrogenism, hyperandrogenism with any adrenal cause, and/or absence of a histological diagnosis.

#### 2.2.3 Data extraction

The titles and abstracts (when available) of the studies retrieved using the described search strategy were screened independently by two review team members (AF and CDL) to identify studies that potentially meet the inclusion criteria. The full texts of potentially eligible studies were retrieved and independently assessed for eligibility by three review team members (DR, RP, and CDA). Selected studies in languages other than English, French, and Italian (i.e., Chinese, Spanish, Portuguese, German, and Japanese) were translated into English or Italian by a specialist translator. Any disagreement over the eligibility of the studies was resolved through discussion between all review team members. A standardized, pre-piloted form was used to extract relevant clinical data from the included studies. The extracted data included: age at PH diagnosis; BMI; ethnicity (4, 5); age of menarche; pregnancy; age of menopause; occurrence of arterial hypertension (AH) and T2DM; levels of T, DHEA-S, 17-OHP and A, FSH, LH, E, SHBG, HOMA-IR, and glycated haemoglobin (HbA1c); the Ferriman–Galway score; occurrence of androgenic alopecia; occurrence of clitoridomegaly; histological diagnosis; follow-up time; and recurrence. Two review team members (AF and CDL) extracted the data independently, and discrepancies were identified and resolved through discussion with DR and RP. When available, missing data were obtained on request from the study authors by mail. The number of patients with data available for the scoping review is reported in **Table 1**. Critical appraisal of the case reports and case series included in the scoping review is reported in **Supplementary Tables S1** and **S2**, respectively (7, 8).

**Table 1:** Clinical and biochemical parameters of patients with ovarian postmenopausal hyperandrogenism surgically treated and classified into tumorous and non-tumorous according to histological diagnosis.

	*N*	Non-tumorous	Tumorous	*p*
Number (%)	281	106 (37.7)	174 (72.3)	
Age at HP diagnosis (years)	273	62.6 (61.4–63.9)	63.1 (61.9–64.3)	0.59
Age at menarche (years)	38	12.4 (11.5–13.2)	13.3 (12.8–13.9)	0.04
Age at menopause (years)	148	49.7 (48.5–50.9)	48.1 (46.9–49.2)	0.048
BMI (kg/m^2^)	138	32.1 (30.9–33.3)	29.6 (28.6–30.6)	<0.01
Ethnicity	97	43	65	0.54
Caucasian	85	39; 90.6	58; 89.2	
Asian	11	4; 9.3	7; 10.8	
Black	1	1; 0.0	0; 0.0	
Ferriman–Gallwey score	127	17.8 (16.2–19.4)	16.3 (14.9–17.7)	0.15
Testosterone (nmol/L)	250	7.3 (5.7–8.9)	18.9 (15.5–22.2)	<0.01
DHEA-S (mmol/L)	153	1.9 (1.5–2.2)	4.1 (2.8–5.3)	0.01
LH (mUI/ml)	160	27.1 (24.0–30.2)	16.4 (13.0–19.8)	<0.01
FSH (mUI/ml)	162	45.4 (41.7–49.1)	29.7 (23.8–35.6)	<0.01
17-OHP (nmol/L)	79	4.0 (2.9–5.0)	6.2 (5.0–7.4)	<0.01
Androstenedione (nmol/L)	55	8.4 (6.2–10.7)	10.6 (8.4–12.9)	0.23
Estradiol (pmol/L)	111	126.3 (84.8–167.4)	215.1 (174.0–256.2)	<0.01

Data are expressed as absolute number, as percentage for dichotomous variables, and as the mean (95% confidence intervals) for continuous variables. *N* is the total number of subjects for whom all data were available.

*BMI*, body mass index; *DHEA-S*, dehydroepiandrosterone sulfate; *FSH*, follicle-stimulating hormone; *LH*, luteinizing hormone; *17-OHP*, 17-hydroxyprogesterone

### 2.3 Statistical analysis

All statistical analyses were performed by LDE using SPSS (Statistical Package for Social Science), version 29 (International Business Machines Corporation, Armonk, NY, USA). Data from the studies selected for the IPD analysis were extracted and included into a single database. These data were then reanalyzed and combined. Continuous variables were compared using analysis of variance for normally distributed data or the Mann–Whitney test for skewed data. The *χ* ^2^ test was used to evaluate differences between categorical variables or proportions. Receiver operating characteristic (ROC) analysis was carried out, and the area under the curve (AUC), with its 95% confidence interval (95%CI), was calculated to evaluate the ability of single clinical characteristics to identify patients with tumorous oPH. Subsequently, the optimal cutoff point of the association was identified using ROC analysis. The ROC analysis was performed for each variable with more than 50 values collected. The risk of specific outcomes was estimated using binary logistic regression analysis and expressed as odds ratio (OR) (95% CI). Moreover, the sensitivity and specificity of the tests were calculated with the following formulas: sensitivity = number of true positives/number of true positives + number of false negatives; specificity = number of true negatives/number of true negatives + number of false positives. Data were expressed as absolute numbers, percentages, or the 50th percentile (25th–75th percentile), as appropriate. A *p*-value <0.05 was considered as statistically significant.

## 3 Results

### 3.1 Case report

**Figure 1:**
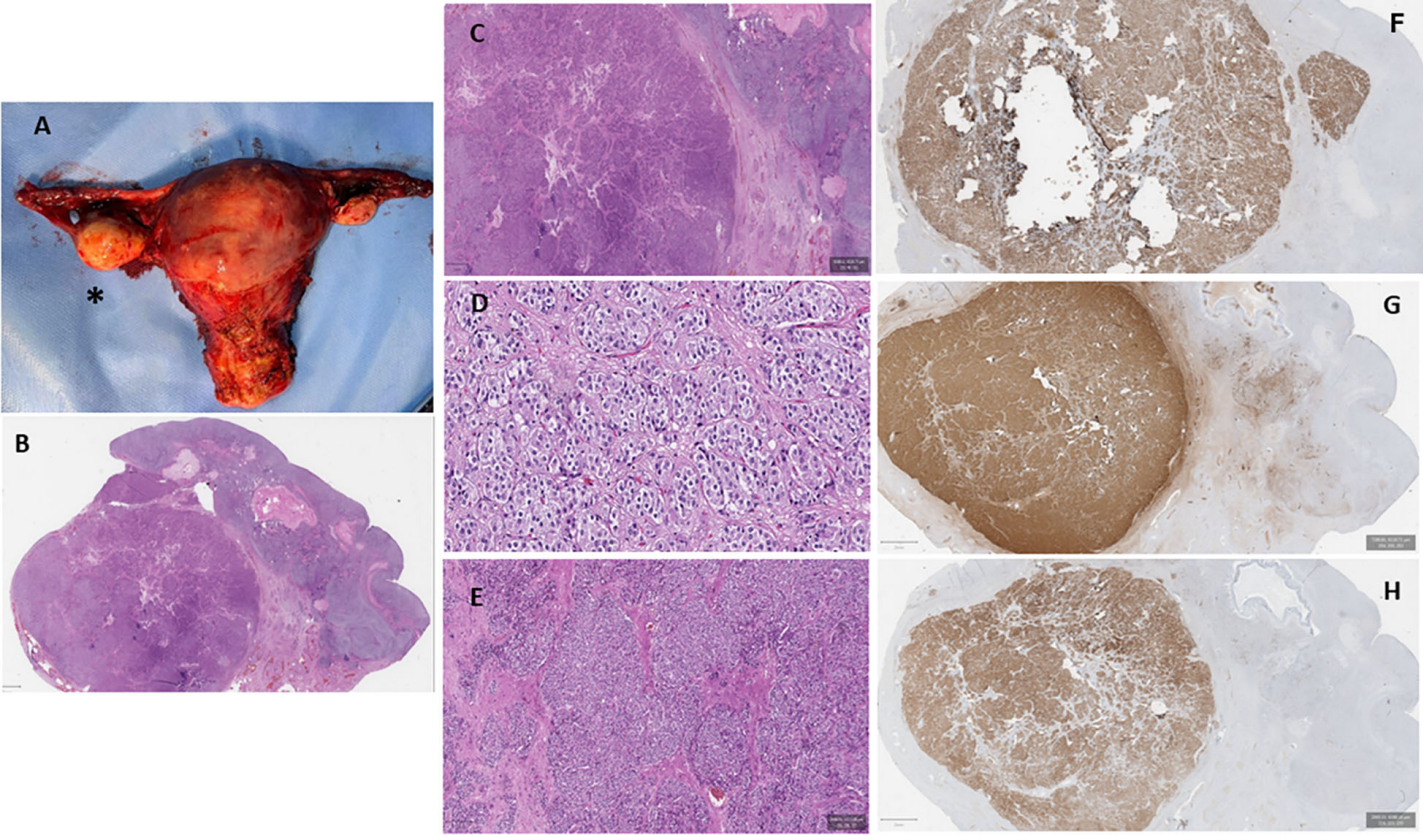
Macroscopic and microscopic appearance of the patient’s internal genitalia. **(A)** Macroscopic appearance of the uterus, the fallopian tubes, and the ovaries. The *asterisk* identifies the right ovary, which appears significantly increased compared with the left ovary. **(B–E)** Ovarian histological examination revealing a well-circumscribed nodular solid tumor composed of groups of large polyhedral, eosinophilic cells with round nuclei and prominent nucleoli [hematoxylin and eosin (H&E) stain: ×2 **(B)** and ×5 **(C)**]. Conspicuous fibrous stroma was present [H&E: ×10 **(D)** and ×20 **(E)**]. **(F–H)** Immunohistochemical evaluation showed strong positivity for melanoma antigen recognized by T1 cells (MART1) **(F)**, calretinin **(G)**, and inhibin B **(H)**.

A postmenopausal 60-year-old woman was referred to the Department of Clinical Medicine and Surgery of the Federico II University in Naples, Italy, for abnormal facial and body hair growth for the past 5 years. She started menstruating at 13 and ended at 50, with regular periods in between. She delivered two male babies through normal vaginal birth. She reported a personal history of T2DM and AH. The physical examination showed androgenic alopecia (second stage according to the Ludwig scale) (9), severe hirsutism (25/36 in the modified Ferriman–Gallwey score) (10), clitoridomegaly (2.5 cm × 3.5 cm) (11), and increased muscle mass. Her BMI was 31.0 kg/m^2^. In **Table 2**, we report the biochemical parameters measured in our PH patient according to the criteria proposed by Hirschberg (12). To obtain reliable reference values, the biochemical parameters of our PH patient were compared to the same measured in 15 controls recruited according to the criteria previously mentioned. As shown in **Table 2**, the circulating levels of androgens, i.e., T, DHEA-S, 17-OHP, and A, measured in our PH patient were higher than the ranges measured in the controls. On the other hand, the FSH and LH levels were lower than the ranges measured in the controls. Serum E, SHBG, HbA1c, and HOMA-IR were withinthe ranges measured in the controls. An adrenocorticotropic hormone (ACTH) 250-µg stimulation test and a dexamethasone 1-mg suppression test were performed according to published criteria, and both tests showed physiological responses (13, 14). A complete blood count (CBC) revealed erythrocytosis, according to the criteria proposed by Babakhanlou et al. (15). Based on the patient’s cardiovascular risk estimated according to the Cardiovascular Risk Chart proposed by the Italian National Institute of Health (16, 17), antiplatelet treatment was prescribed. Pelvic transabdominal ultrasound examination was normal, whereas the transvaginal ultrasonography (TVU) showed the right ovary increasing by size according to age. Pelvic computed tomography (CT) and magnetic resonance (MR) confirmed the increased size of the right ovary. Based on the biochemical and instrumental data, the patient underwent robotic hysterectomy with bilateral ovariectomy (18). Macroscopically, the right ovary was larger than the left (right ovary, 30 mm × 20 mm; left ovary, 20 mm × 10 mm) (**Figure 1A**). Histological examination showed a Leydig cell tumor (**Figures 1B–E**) (19). The patient was classified as stage 1A according to the International Federation of Gynecology and Obstetrics (FIGO) criteria (19). As reported in **Table 2**, 1 week after surgery, all of the biochemical parameters measured in our PH patient returned to normal values, as compared with the controls. The erythrocytosis resolved in 8 weeks, and antiplatelet therapy was dismissed. After 6 months, clinical examination of the patients no longer showed clinical signs of hyperandrogenism.

**Table 2:** Biochemical parameters of the patients at enrollment and after surgery.

Biochemical parameter	Before surgery	After surgery	Reference value
FSH (mUI/ml)	42.3	47.6	72.2 (53.7–90.7)
LH (mUI/ml)	18.9	25.2	27.9 (18.6–37.3)
Total testosterone (nmol/l)	18	0.51	0.6 (0.3–0.9)
SHBG (mg/L)	13.4	13.2	13.5 (12.9–14.1)
Androstenedione (nmol/L)	5.68	1.18	0.83 (0.30–1.63)
Dhea-s (mmol/L)	1.53	2.62	1.8 (0.7–2.9)
Estradiol (pmol/L)	88.10	58.74	81.9 (47.4–116.3)
17-OHP (nmol/L)	6.69	1.35	1.3 (0.3–2.3)
Inhibin B (U/ml)	<0.4	<0.4	<0.4
HOMA-IR	3.56	3.40	3.21 (2.81–3.62)
HbA1c (%)	6.7	6.6	6.7 (6.5–6.9)
24h free urinary cortisol (nmol/24h)	37.80	34.10	35.7 (31.7–39.7)

References values were collected from 15 postmenopausal women and expressed as the mean (95% confidence intervals).

FSH, follicle-stimulating hormone; LH, luteinizing hormone; SHBG, sex hormone-binding globulin; Dhea-s, dehydroepiandrosterone sulfate; 17-OHP, 17-OH progesterone; HOMA-IR, homeostatic model assessment of insulin resistance; HbA1c, glycated hemoglobin.

### 3.2 Scoping review

**Figure 2:**
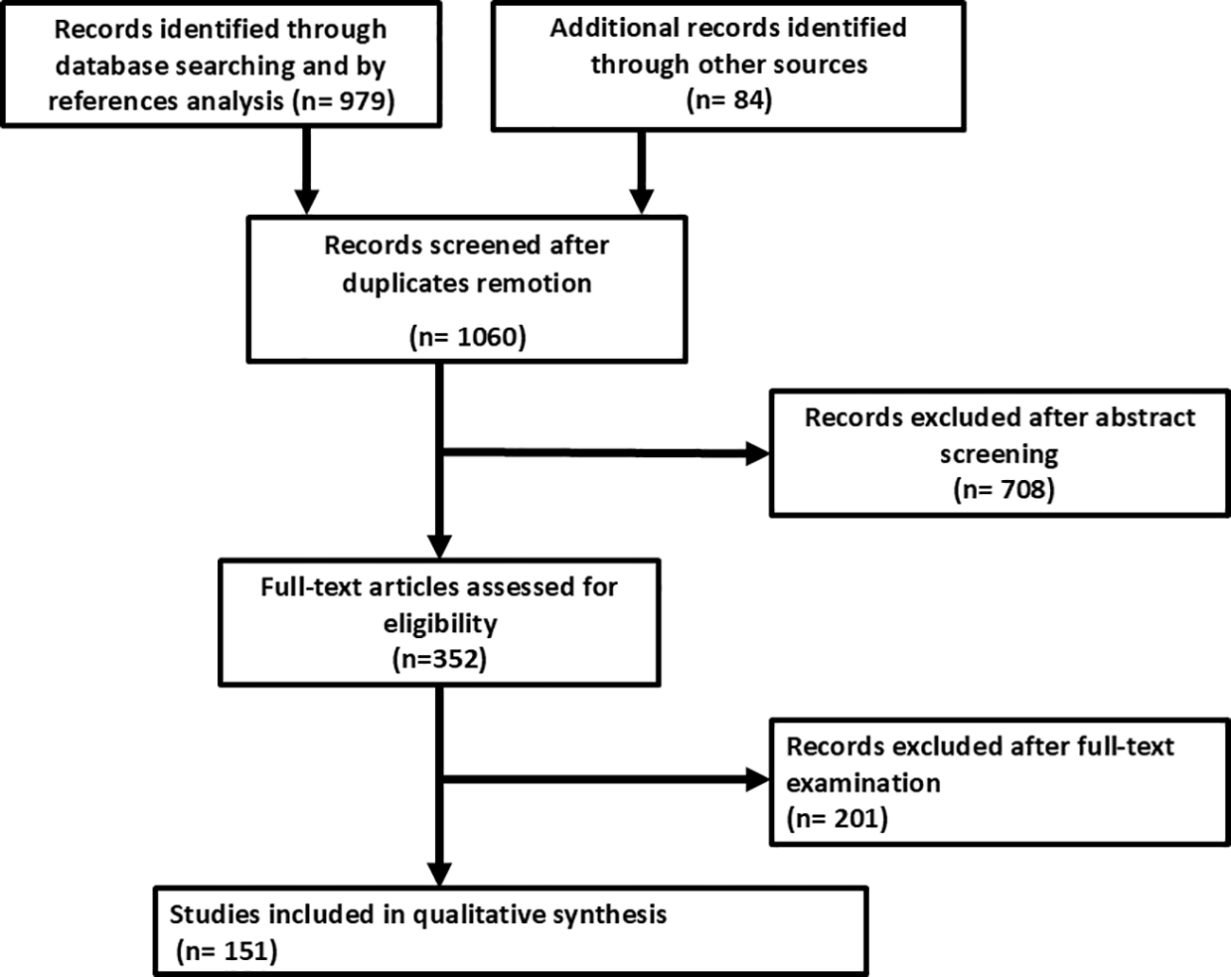
PRISMA flowchart.

As reported in **Figure 2**, the search strategy identified 1,060 studies after removal of duplicates. After the exclusion of the studies that did not meet the inclusion criteria, 151 studies were finally included in the qualitative and quantitative syntheses. There were 123 case reports and 28 case series included in the final analysis. The complete list of studies included in the scoping review with IPD analysis is shown in **Supplementary Tables S1** and **S2** (7, 8).

Overall, 280 patients with oPH were included in the IPD analysis. According to the criteria proposed by Markopoulos et al. (3), 280 patients with oPH were dichotomized according to the histological diagnosis: 174 tumorous oPH and 106 non-tumorous oPH patients (**Table 1**).

According to the World Health Organization criteria (20), among the patients with tumorous oPH, 127 (72.9%) [mean age = 62.8 (61.4–64.2) years, mean BMI = 29.3 (28.2–30.9) kg/m^2^] were affected by pure stromal tumors, 14 (8%) [mean age = 62.9 (56.5–69.3) years, mean BMI = 31.1 (24.0–38.1) kg/m^2^] by epithelial tumors, 11 (6.3%) [mean age = 63.0 (56.4–69.5) years; BMI = 26.0 kg/m^2^] affected by mixed sex cord stromal tumors, 8 (4.6%) [mean age = 64.2 (50.6–77.7) years; mean BMI = 30.3 (24.0–36.5) kg/m^2^] by pure sex cord tumors, 3 (1.7%) [mean age = 70 (55–78) years] by germ cell tumors, and 11 (6.3%) [mean age = 67.0 (53.6–80.4) years] affected by not specified virilizing ovarian tumor (VOT). Among the patients with non-tumorous oPH, 94 (88.7%) [mean age = 62.4 (61.1–63.8) years, mean BMI = 32.2 (30.9–33.5) kg/m^2^] were affected by ovarian hyperthecosis and the remaining 12 (11.3%) [mean age = 63.3 (59.1–67.1) years, mean BMI = 30.4 (25.1–35.7) kg/m^2^] by ovarian Leydig cell hyperplasia. Follow-up information was available for all oPH patients. The follow-up period after surgery was 3 (1–24) months. Data analyses indicated that no patient showed PH recurrence after surgery, i.e., curative in all cases.

#### 3.2.1 Biochemical characteristics of oPH patients

**Figure 3:**
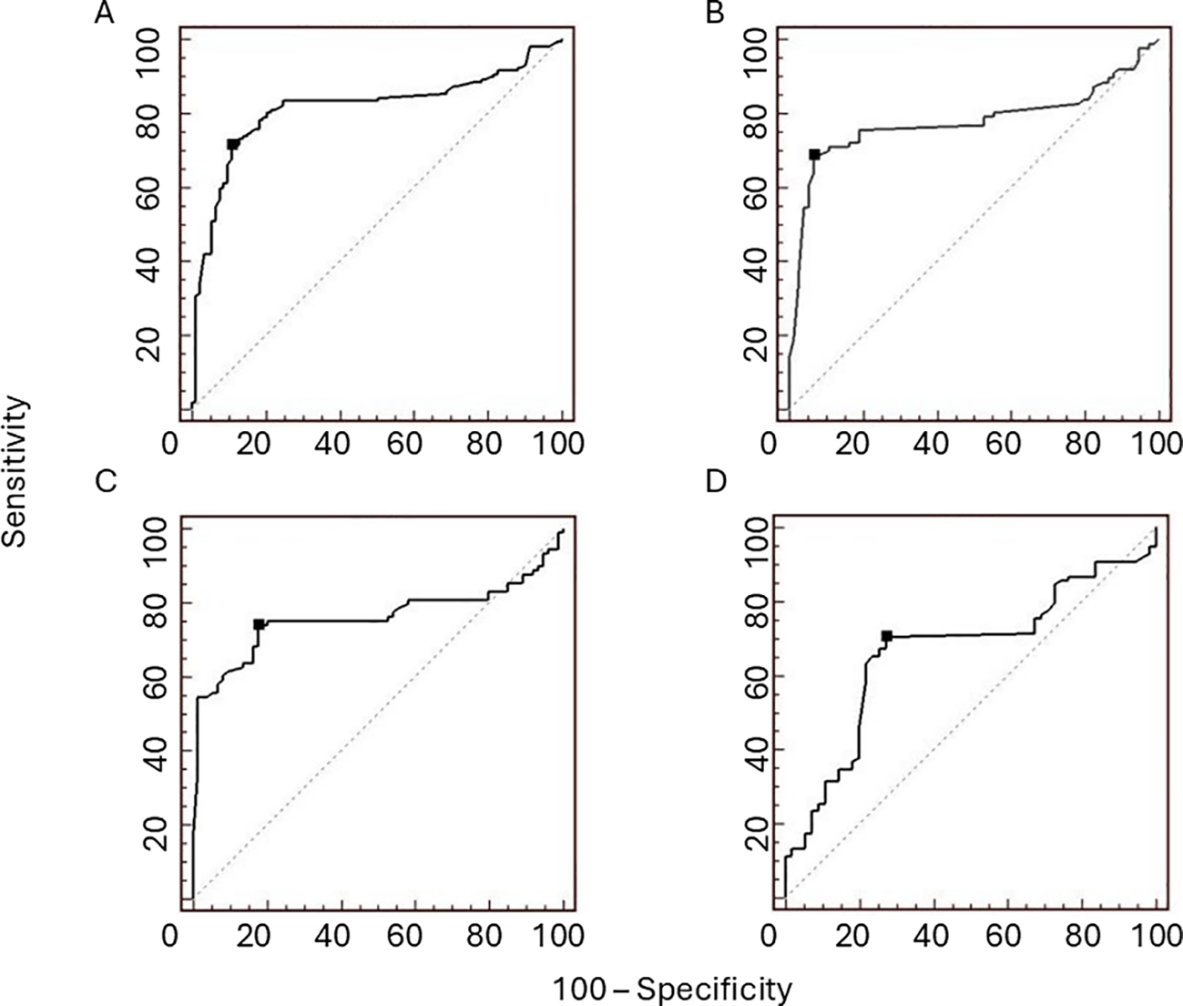
Receiver operating characteristic (ROC) curves for testosterone (T), luteinizing hormone (LH), follicle-stimulating hormone (FSH), and dehydroepiandrosterone sulfate (DHEA-S) in patients with ovarian postmenopausal hyperandrogenism (PH). The areas under curve (AUCs) of T **(A)**, LH **(B)**, FSH **(C)**, and DHEA-S **(D)** are 0.88 (0.76–0.86), 0.78 (0.70–0.84), 0.76 (0.69–0.82), and 0.66 (0.58–0.74), respectively. The proposed cutoff points (*black dots*) for differentiating ovarian PH patients with tumours from ovarian PH patients without tumours, with suitable sensitivity and specificity, are T ≥9.8 nmol/L (sensitivity = 71.3%, specificity = 89.2%), LH ≤15 mUI/ml (sensitivity = 68.6%, specificity = 93.2%), FSH ≤35 mUI/ml (sensitivity = 73.9%, specificity = 82.4%), and DHEA-S ≥1.6 μmol/L (sensitivity = 70.5%, specificity = 72.7%).

As shown in **Table 1**, patients with tumorous oPH showed lower BMI compared with those with non-tumorous oPH. Regarding the biochemical parameters, patients with tumorous oPH showed lower levels of LH and FSH and higher levels of T, DHEA-S, 17-OHP, and E compared with non-tumorous oPH patients (*p* < 0.05 in all cases) (**Table 1**). According to previously published criteria, ROC analysis was performed for T, LH, FSH, and DHEA-S. As shown in **Figures 3A–D**, the AUCs of T, LH, FSH, and DHEA-S were 0.88 (0.76–0.86), 0.78 (0.70–0.84), 0.76 (0.69–0.82), and 0.66 (0.58–0.74), respectively. Consecutively, we suggest the following cutoff points for the identification of tumorous oPH: T ≥ 9.8 nmol/L (sensitivity = 71.3%, specificity = 89.2%), LH ≤ 15 mUI/ml (sensitivity = 68.6%, specificity = 93.2%), FSH ≤ 35 mUI/ml (sensitivity = 73.9%, specificity = 82.4%), and DHEA-S ≥ 1.6 μmol/L (sensitivity = 70.5%, specificity = 72.7%).

#### 3.2.2 Radiological examination of oPH patients

A total of 260 (92.5%) patients underwent pelvis radiological examinations during the oPH diagnostic process. As reported in **Table 3**, 247 patients with oPH underwent a TVU, 139 a pelvic CT scan, and 107 a pelvic magnetic resonance imaging (MRI). Overall, the pelvic radiological examination identified the ovarian condition that caused PH in 176 patients (59 patients affected by non-tumorous oPH and 117 affected by tumorous oPH). In the case of a radiological finding of macroscopic abnormalities (i.e., increased volume of an ovary and/or abnormal age-corrected ovary size), the OR of tumorous oPH was 2.08 (95%CI = 1.22–3.53, *p* < 0.01). The sensitivity and specificity of TVU, CT, and MRI for oPH are reported in **Table 3**.

**Table 3:** Sensitivity and specificity of the radiological methodology in surgically treated ovarian postmenopausal hyperandrogenism (oPH) patients.

Methodology	*N*	Sensitivity	Specificity
TVU	247	49%	42%
CT	139	48%	87%
MRI	107	68%	36%

*N* is the number of patients with oHP who underwent each procedure.

TVU, transvaginal ultrasonography; CT, computerized tomography; MRI, magnetic resonance imaging.

## 4 Discussion

PH is a rare condition that requires a careful and laborious differential diagnosis, considering that it may be dependent on the neoplastic source of androgens. The results of this study provide clinical and biochemical parameters for the identification of tumorous oPH before the surgical results. Indeed, patients with tumorous oPH show lower BMI, LH, and FSH and higher T, E, and DHEA-S compared with non-tumorous oPH patients. Based on the data collected using IPD, we proposed cutoff points for T, DHEA-S, LH, and FSH for the identification of patients with tumorous oPH with good sensitivity and specificity. The female reproduction system is physiologically regulated by intricate neuroendocrine signals involving the hypothalamic–pituitary–ovarian (HPO) axis. Overweight and obesity can disturb the HPO axis, causing metabolic and reproductive disorders, as observed in patients with non-tumorous oPH (21). According to our results, we hypothesize that the interference of adiposity on the HPO axis, mediated by insulin and leptin resistance and by low-grade chronic inflammation (LGCI), is responsible for the occurrence of PH in the case of non-tumorous ovarian conditions (22). On the contrary, patients with tumorous oPH showed simultaneous increases in T and DHEA-S, which can reduce the gonadotropin levels even in the postmenopausal era, as observed in our data collection. The increased DHEA-S levels may be related to the hyper-expression of steroid sulfatase androgen-activating enzymes in tumorous ovarian cells (23, 24). As shown in **Table 3**, the IPD analysis also indicated that all of the radiologic procedures showed good sensitivity and specificity in identifying ovarian diseases behind oPH. Based on these data and according to Hofland et al. (25), we suggest performing a pelvic exploratory laparotomy after the exclusion of the adrenal PH causes, if any radiological pelvic examination is not conclusive. However, an ovarian cancer causing PH is very likely in cases where ovarian macroscopic abnormalities are reported. No recurrence was reported in oPH patients after surgery.

This study has strengths and limitations. The IPD procedure guarantees the best opportunity to summarize the results of multiple case studies and case series, offering a large amount of data in the case of rare conditions (26, 27). Furthermore, IPD analysis allows the evaluation of the predictive value of multiple individual variables, such as, in patients with PH, the T, DHEA-S, LH, and FSH levels, and the standardization of the statistical analysis (28, 29). On the other hand, the heterogeneity of the data, the lack of centralized measurements of the instrumental and biochemical parameters, and the missing information with regard to the assay methods are considered limitations because these do not ensure uneven data. Lastly, one other limit is represented by the selected inclusion and exclusion criteria, which are restricted to a subset of HP patients with ovarian diseases requiring a surgical approach. Indeed, the surgical indication suggests a more severe hyperandrogenism and/or an imaging suspicion of ovarian abnormality, so that just a subset of women with PH were included (operated oPH women with a conclusive histological diagnosis), impairing the generalizability of the results.

## 6 Conclusion

PH is a rare clinical condition that requires long and delicate diagnostic procedures. For the first time, the current study provided an interpretation of some commonly used biochemical parameters to properly address the diagnostic assessment of oPH. In particular, lower BMI, LH, and FSH and higher T, E, and DHEA-S can be used to distinguish tumorous oPH.

## Data Availability

The original contributions presented in the study are included in the article/**Supplementary Material**. Further inquiries can be directed to the corresponding authors.
