# Supplementary material for: Postmenopausal ovarian hyperandrogenism of surgically treated patients: a case report and scoping review with individual patient’s data analysis
Source: Front Endocrinol (Lausanne). 2025 Aug 1;16:1495930. doi: 10.3389/fendo.2025.1495930 (PMC12353694; doi:10.3389/fendo.2025.1495930)
Supplement: Supplementary file 1 [file Table1.docx]

Supplementary table 1

**Quality assessment of case reports included in the systematic review according to Joanna Briggs Institute Critical Appraisal Checklist for Case Reports^#^**

|  | **Question** | | | | | | | |  |
| --- | --- | --- | --- | --- | --- | --- | --- | --- | --- |
| **Reference** | **1** | **2** | **3** | **4** | **5** | **6** | **7** | **8** | |
|  | Y | Y | Y | Y | Y | Y | NA | N | |
|  | Y | Y | Y | Y | Y | Y | NA | N | |
|  | Y | Y | Y | Y | Y | Y | NA | N | |
|  | Y | Y | Y | Y | Y | Y | NA | N | |
|  | Y | Y | Y | Y | Y | Y | NA | N | |
|  | Y | Y | Y | Y | Y | Y | NA | N | |
|  | Y | Y | Y | Y | Y | Y | NA | N | |
|  | Y | Y | Y | Y | Y | Y | NA | N | |
|  | Y | Y | Y | Y | Y | Y | NA | N | |
|  | Y | Y | Y | Y | Y | Y | NA | N | |
|  | Y | Y | Y | Y | Y | Y | NA | N | |
|  | Y | Y | Y | Y | Y | Y | NA | N | |
|  | Y | Y | Y | Y | Y | Y | NA | N | |
|  | Y | Y | Y | Y | Y | Y | NA | N | |
|  | Y | Y | Y | Y | Y | Y | NA | N | |
|  | Y | Y | Y | Y | Y | Y | NA | N | |
|  | Y | Y | Y | Y | Y | Y | NA | N | |
|  | Y | Y | Y | Y | Y | Y | NA | N | |
|  | Y | Y | Y | Y | Y | Y | NA | N | |
|  | Y | Y | Y | Y | Y | Y | NA | N | |
|  | Y | Y | Y | Y | Y | Y | NA | N | |
|  | Y | Y | Y | Y | Y | Y | NA | N | |
|  | Y | Y | Y | Y | Y | Y | NA | N | |
|  | Y | Y | Y | Y | Y | Y | NA | N | |
|  | Y | Y | Y | Y | Y | Y | NA | N | |
|  | Y | Y | Y | Y | Y | Y | NA | N | |
|  | Y | Y | Y | Y | Y | Y | NA | N | |
|  | Y | Y | Y | Y | Y | Y | NA | N | |
|  | Y | Y | Y | Y | Y | Y | NA | N | |
|  | Y | Y | Y | Y | Y | Y | NA | N | |
|  | Y | Y | Y | Y | Y | Y | NA | N | |
|  | Y | Y | Y | Y | Y | Y | NA | N | |
|  | Y | Y | Y | Y | Y | Y | NA | N | |
|  | Y | Y | Y | Y | Y | Y | NA | Y | |
|  | Y | Y | Y | Y | Y | Y | NA | Y | |
|  | Y | Y | Y | Y | U | N | NA | Y | |
|  | Y | Y | Y | Y | Y | Y | NA | Y | |
|  | Y | Y | Y | Y | Y | Y | NA | Y | |
|  | Y | Y | Y | Y | Y | Y | NA | Y | |
|  | Y | Y | Y | Y | Y | Y | NA | Y | |
|  | Y | Y | Y | Y | Y | Y | NA | N | |
|  | Y | Y | Y | Y | Y | Y | NA | Y | |
|  | Y | Y | Y | Y | Y | Y | NA | Y | |
|  | Y | Y | Y | Y | Y | Y | NA | N | |
|  | Y | Y | Y | Y | Y | Y | NA | N | |
|  | Y | Y | Y | Y | Y | Y | NA | N | |
|  | Y | Y | Y | Y | Y | Y | Y | Y | |
|  | Y | Y | Y | Y | Y | Y | NA | N | |
|  | Y | Y | Y | Y | Y | Y | Y | N | |
|  | Y | Y | Y | Y | Y | Y | NA | N | |
|  | N | N | N | Y | N | N | NA | N | |
|  | Y | Y | Y | Y | Y | Y | NA | N | |
|  | Y | Y | Y | Y | Y | Y | NA | N | |
|  | Y | Y | Y | Y | Y | Y | NA | Y | |
|  | Y | Y | Y | Y | Y | Y | NA | N | |
|  | Y | Y | Y | Y | Y | Y | NA | N | |
|  | Y | Y | Y | Y | Y | Y | NA | N | |
|  | N | N | Y | Y | Y | Y | Na | N | |
|  | Y | Y | Y | Y | Y | Y | NA | N | |
|  | Y | Y | Y | Y | Y | Y | NA | N | |
|  | Y | Y | Y | Y | Y | Y | NA | N | |
|  | Y | N | Y | Y | N | Y | NA | N | |
|  | Y | Y | Y | Y | Y | Y | NA | N | |
|  | N | N | Y | Y | Y | Y | NA | N | |
|  | Y | Y | Y | Y | Y | Y | NA | N | |
|  | Y | Y | Y | Y | Y | Y | NA | N | |
|  | Y | Y | Y | Y | Y | Y | NA | N | |
|  | Y | Y | Y | Y | Y | Y | NA | N | |
|  | Y | Y | Y | Y | Y | Y | NA | N | |
|  | Y | Y | Y | Y | Y | Y | NA | N | |
|  | Y | Y | Y | Y | Y | Y | NA | N | |
|  | Y | Y | Y | Y | Y | Y | NA | N | |
|  | Y | Y | Y | Y | Y | Y | NA | N | |
|  | Y | Y | Y | Y | Y | Y | NA | N | |
|  | Y | Y | Y | Y | Y | Y | NA | N | |
|  | Y | Y | Y | Y | Y | Y | NA | N | |
|  | Y | Y | Y | Y | Y | Y | NA | N | |
|  | Y | Y | Y | Y | Y | Y | NA | N | |
|  | Y | Y | Y | Y | Y | Y | NA | N | |
|  | Y | Y | Y | Y | Y | Y | NA | N | |
|  | Y | Y | Y | Y | Y | Y | NA | N | |
|  | Y | Y | Y | Y | Y | Y | NA | N | |
|  | Y | Y | Y | Y | Y | Y | NA | N | |
|  | Y | Y | Y | Y | Y | Y | NA | N | |
|  | Y | Y | Y | Y | Y | Y | NA | N | |
|  | Y | Y | Y | Y | Y | Y | NA | N | |
|  | Y | Y | Y | Y | Y | Y | NA | N | |
|  | Y | Y | Y | Y | Y | Y | NA | N | |
|  | Y | Y | Y | Y | Y | Y | NA | N | |
|  | Y | Y | Y | Y | Y | Y | NA | N | |
|  | Y | Y | Y | Y | Y | Y | NA | N | |
|  | Y | Y | Y | Y | Y | Y | NA | N | |
|  | Y | Y | Y | Y | Y | Y | NA | N | |
|  | Y | Y | Y | Y | Y | Y | NA | N | |
|  | Y | Y | Y | Y | Y | Y | NA | N | |
|  | Y | Y | Y | Y | Y | Y | NA | N | |
|  | Y | Y | Y | Y | Y | Y | NA | N | |
|  | Y | Y | Y | Y | Y | Y | NA | N | |
|  | Y | Y | Y | Y | Y | Y | NA | N | |
|  | Y | Y | Y | Y | Y | Y | NA | N | |
|  | Y | Y | Y | Y | Y | Y | NA | N | |
|  | Y | Y | Y | Y | Y | Y | NA | N | |
|  | Y | Y | Y | Y | Y | Y | NA | N | |
|  | Y | Y | Y | Y | Y | Y | NA | N | |
|  | Y | Y | Y | Y | Y | Y | NA | N | |
|  | Y | Y | Y | Y | Y | Y | NA | N | |
|  | Y | Y | Y | Y | Y | Y | NA | N | |
|  | Y | Y | Y | Y | Y | Y | NA | N | |
|  | Y | Y | Y | Y | Y | Y | NA | N | |
|  | Y | Y | Y | Y | Y | Y | NA | N | |
|  | Y | Y | Y | Y | Y | Y | NA | N | |
|  | Y | Y | Y | Y | Y | Y | NA | N | |
|  | Y | Y | Y | Y | Y | Y | NA | N | |
|  | Y | Y | Y | Y | Y | Y | NA | N | |
|  | Y | Y | Y | Y | Y | Y | NA | N | |
|  | Y | Y | Y | Y | Y | Y | NA | N | |
|  | Y | Y | Y | Y | Y | Y | NA | N | |
|  | Y | Y | Y | Y | Y | Y | NA | N | |
|  | Y | Y | Y | Y | Y | Y | NA | N | |
|  | Y | Y | Y | Y | Y | Y | NA | N | |
|  | Y | Y | Y | Y | Y | Y | NA | N | |
|  | Y | Y | Y | Y | Y | Y | NA | N | |
|  | Y | Y | Y | Y | Y | Y | NA | N | |

1. Were patient’s demographic characteristics clearly described?
2. Was the patient’s history clearly described and presented as a timeline?
3. Was the current clinical condition of the patient on presentation clearly described?
4. Were diagnostic tests or assessment methods and the results clearly described?
5. Was the intervention(s) or treatment procedure(s) clearly described?
6. Was the post-intervention clinical condition clearly described?
7. Were adverse events (harms) or unanticipated events identified and described?
8. Does the case report provide takeaway lessons?

**Yes: Y/No: N/Unclear: U/Not applicable: N.A**.

# Moola S, Munn Z, Tufanaru C, Aromataris E, Sears K, Sfetcu R, Currie M, Lisy K, Qureshi R, Mattis P, Mu P. Chapter 7: Systematic reviews of etiology and risk. In: Aromataris E, Munn Z (Editors)*. JBI Manual for Evidence Synthesis.* JBI, 2020. Available from https://synthesismanual.jbi.global. https://doi.org/10.46658/JBIMES-20-08

**CASE REPORTS REFERENCES**

1. Lal RA, Basina M. Postmenopausal Hyperandrogenism. J Womens Health Care. 2018;7(1):e132. doi: 10.4172/2167-0420.1000e132.
2. De Vis M, Brock S, Cosyns S, Velkeniers B. A rare cause of postmenopausal hyperandrogenism. BMJ Case Rep. 2021 Jan 7;14(1):e237505. doi: 10.1136/bcr-2020-237505.
3. Roque JJN, Alves IBS, Rodrigues AMAPF, Bugalho MJ. A benign cause of hyperandrogenism in a postmenopausal woman. Endocrinol Diabetes Metab Case Rep. 2021 Feb 17;2021:20-0054. doi: 10.1530/EDM-20-0054.
4. Lozoya Araque T, Monfort Ortiz IR, Martín González JE, Jiménez García A, Navarro Hidalgo I, Andrade Gamarra V, Parrell Soler C, Gil Raga F. Ovarian Stromal Hyperplasia: A Rare Cause of Postmenopausal Hyperandrogenism. J Menopausal Med. 2020 Apr;26(1):39-43. doi: 10.6118/jmm.19012.
5. Castro-Dufourny I, Saiz-Pardo-Sanz M. Hiperandrogenismo tras la menopausia [Hyperandrogenism in a postmenopausal woman]. Rev Clin Esp (Barc). 2013 Apr;213(3):e19-22. Spanish. doi: 10.1016/j.rce.2012.12.004.
6. Shearer JL, Salmons N, Murphy DJ, Gama R. Postmenopausal hyperandrogenism: the under-recognized value of inhibins. Ann Clin Biochem. 2017 Jan;54(1):174-177. doi: 10.1177/0004563216656873.
7. De Taddeo S, Andreadi A, Minasi A, D'Ippolito I, Borelli B, Meloni M, Romano M, Ruotolo V, Cacciotti L, Rizzo G, Patrizi L, Bellia A, Lauro D. Surgical treatment of post-menopausal ovarian hyperandrogenism improves glucometabolic profile alongside clinical hirsutism. SAGE Open Med Case Rep. 2023 Jun 5;11:2050313X231178404.
8. Karangadan S, Nair IR, Menon U, Bhati P, Remadevi C. How hairfall saved the patient from cancer twice - A postmenopausal patient with synchronous endometrial and endocervical carcinomas presenting with endocrine symptoms. Post Reprod Health. 2023 Jun;29(2):109-112. doi: 10.1177/20533691231167660.
9. Metzker LS, Ferreira LAC, Borges JCN, Guzzo MF, Ferreira RN, Silva LLR, Cavedo RM, Filho AC. Postmenopausal Hyperandrogenism due to Ovarian Hyperthecosis. Case Rep Obstet Gynecol. 2023 Jan 27;2023:2783464. doi: 10.1155/2023/2783464.
10. Wu YC, Zhang JC, Xu KH, Zhong Q. Postmenopausal Hyperandrogenism with Undetectable Origin. J Minim Invasive Gynecol. 2023 Apr;30(4):251-253. doi: 10.1016/j.jmig.2022.12.020.
11. Hirschberg AL. Approach to Investigation of Hyperandrogenism in a Postmenopausal Woman. J Clin Endocrinol Metab. 2023 Apr 13;108(5):1243-1253. doi: 10.1210/clinem/dgac673
12. Khalid A, Kotha M, Munir AA. Rare case of testosterone producing serous cystadenoma. Oxf Med Case Reports. 2022 Aug 18;2022(8):omac087. doi: 10.1093/omcr/omac087.
13. Suturina LV, Sharifulin EM, Sharifulin MA, Lazareva LM, Danusevich IN, Ievleva KD, Nadeliaeva IG. The Leydig Steroid Cell Tumor in a Postmenopausal Woman with Clinical and Biochemical Hyperandrogenism: A Case Report. Metabolites. 2022 Jul 4;12(7):620. doi: 10.3390/metabo12070620.
14. Sumanatilleke M, de Silva NL, Ranaweera G, Appuhamy C, Karunaratne K, de Silva MVC. Postmenopausal hyperandrogenism due to an ovarian sex cord-stromal tumour causing elevated dehydroepiandrosterone sulphate: a case report. BMC Womens Health. 2022 Jul 17;22(1):297. doi: 10.1186/s12905-022-01879-8.
15. Johnson JE, Hussain M, Rathore A, Wolfe K. A rare case of postmenopausal hyperandrogenism due to ovarian hyperthecosis and hilus cell hyperplasia. Post Reprod Health. 2022 Mar;28(1):51-55. doi: 10.1177/20533691211073451.
16. Mamoojee Y, Ganguri M, Taylor N, Quinton R. Clinical Case Seminar: Postmenopausal androgen excess-challenges in diagnostic work-up and management of ovarian thecosis. Clin Endocrinol (Oxf). 2018 Jan;88(1):13-20. doi: 10.1111/cen.13492.
17. Alpañés M, González-Casbas JM, Sánchez J, Pián H, Escobar-Morreale HF. Management of postmenopausal virilization. J Clin Endocrinol Metab. 2012 Aug;97(8):2584-8. doi: 10.1210/jc.2012-1683.
18. Gandrapu B, Sundar P, Phillips B. Hyperandrogenism in a Postmenopausal Woman Secondary to Testosterone Secreting Ovarian Stromal Tumor with Acoustic Schwannoma. Case Rep Endocrinol. 2018 Dec 5;2018:8154513. doi: 10.1155/2018/8154513.
19. Guarino A, Di Benedetto L, Giovanale V, Rampioni Vinciguerra GL, Stoppacciaro A, Bellati F, Caserta D. Hyperandrogenism in a postmenopausal woman: a rare case of ectopic adrenal cortical gland. Gynecol Endocrinol. 2017 Mar;33(3):185-187. doi: 10.1080/09513590.2016.1252326.
20. 21.Hussain A, Uy E, Marlowe S, Piercy J, Akbar A. A Rare Case of Hyperandrogenism Due to Fibrothecoma and Leydig Cell Tumor in a Postmenopausal Woman With Adrenal Adenoma: A Case Report and Literature Review. Cureus. 2023 Aug 9;15(8):e43180. doi: 10.7759/cureus.43180.
21. García E, García-Hierro V, De La Maza L, Alvarez P, Santos E, Pi J, Castillo L, Ruiz E. Hyperandrogenism in a postmenopausal woman. Endocrinol Nutr. 2008 Oct;55(8):376-8. English, Spanish. doi: 10.1016/S1575-0922(08)72800-6.
22. Czyzyk A, Latacz J, Filipowicz D, Podfigurna A, Moszynski R, Jasinski P, Sajdak S, Gaca M, Genazzani AR, Meczekalski B. Severe hyperandrogenemia in postmenopausal woman as a presentation of ovarian hyperthecosis. Case report and mini review of the literature. Gynecol Endocrinol. 2017 Nov;33(11):836-839. doi: 10.1080/09513590.2017.1337094.
23. Rajamani K, Moore RG, Stanard SM, Astapova O. Testosterone-Secreting Endometrioid Ovarian Carcinoma Presenting With Hyperandrogenism. AACE Clin Case Rep. 2022 Jan 25;8(3):135-138. doi: 10.1016/j.aace.2022.01.003
24. Lambrinoudaki I, Dafnios N, Kondi-Pafiti A, Triantafyllou N, Karopoulou E, Papageorgiou A, Augoulea A, Armeni E, Creatsa M, Vlahos N. A case of postmenopausal androgen excess. Gynecol Endocrinol. 2015 Oct;31(10):760-4. doi: 10.3109/09513590.2015.1075500.
25. Bachelot A, Meduri G, Baudin E, Kuttenn F, Touraine P. Hyperandrogenism in a postmenopausal woman presenting with a metastatic ileum endocrine tumor. Fertil Steril. 2004 Mar;81(3):675-8. doi: 10.1016/j.fertnstert.2003.07.040.
26. Chen M, Zhou W, Zhang Z, Zou Y, Li C. An ovarian Leydig cell tumor of ultrasound negative in a postmenopausal woman with hirsutism and hyperandrogenism: A case report. Medicine (Baltimore). 2018 Mar;97(10):e0093. doi: 10.1097/MD.0000000000010093.
27. Pinto AM, Martins MB, Oliveira N, Oliveira M. Ovarian steroid cell tumour inducing virilisation in a postmenopausal woman. BMJ Case Rep. 2022 Apr 20;15(4):e249907. doi: 10.1136/bcr-2022-249907.
28. Pathmanathan S, De Silva SDN, Sumanatilleke M, Lokuhetty D, Ranathunga UVV. Bilateral Leydig Cell Hyperplasia: A Rare Cause of Postmenopausal Hirsutism. Case Rep Endocrinol. 2022 Feb 12;2022:8804856. doi: 10.1155/2022/8804856.
29. Koeneman MM, Heiligers-Duckers C, van der Velde R, Wouda S, de Rooij MJ, Janssen MJ, Boskamp D. Ovarian Leydig cell hyperplasia as a rare cause of hair loss in a postmenopausal female patient: a case report and diagnostic approach toward postmenopausal hyperandrogenism. Eur J Obstet Gynecol Reprod Biol. 2016 Apr;199:198-200. doi: 10.1016/j.ejogrb.2016.01.024.
30. Vatopoulou A, Gkrozou F, Birbas E, Kanavos T, Skentou C, Miliaras D. Leydig cell hyperplasia as a cause of virilization in a postmenopausal woman: A case report. Case Rep Womens Health. 2023 Aug 29;39:e00537. doi: 10.1016/j.crwh.2023.e00537.
31. Taylor HC, Pillay I, Setrakian S. Diffuse stromal Leydig cell hyperplasia: a unique cause of postmenopausal hyperandrogenism and virilization. Mayo Clin Proc. 2000 Mar;75(3):288-92. doi: 10.4065/75.3.288.
32. Benavent Correro P, Sáenz Valls M, García Cano A, Jiménez Mendiguchia L, Moreno Moreno E, Luque-Ramírez M. An unusual circulating steroid profile in a virilized postmenopausal woman. Diagnosis (Berl). 2018 Jun 27;5(2):83-87. doi: 10.1515/dx-2018-0007.
33. Bužinskienė D, Marčiukaitytė R, Šidlovska E, Rudaitis V. Ovarian Leydig Cell Tumor and Ovarian Hyperthecosis in a Postmenopausal Woman: A Case Report and Literature Review. Medicina (Kaunas). 2023 Jun 6;59(6):1097. doi: 10.3390/medicina59061097.
34. Larrea AL, González VR, Knoblovits P, Gil SJ. Virilización y enfermedad metabólica en mujer postmenopáusica relacionado a hiperplasia e hipertecosis ovárica [Virilization and metabolic disease in postmenopausal women related to ovarian hyperplasia and hypertecosis]. Rev Fac Cien Med Univ Nac Cordoba. 2021 Jun 28;78(2):193-196. Spanish. doi: 10.31053/1853.0605.v78.n2.32136.
35. J Orrego J, A Chorny J. Aldosterone- and cortisol-cosecreting adrenal adenoma, ovarian hyperthecosis and breast cancer. Endocrinol Diabetes Metab Case Rep. 2020 Sep 23;2020:20-0121. doi: 10.1530/EDM-20-0121.
36. Souto SB, Baptista PV, Braga DC, Carvalho D. Ovarian Leydig cell tumor in a post-menopausal patient with severe hyperandrogenism. Arq Bras Endocrinol Metabol. 2014 Feb;58(1):68-75. doi: 10.1590/0004-2730000002461.
37. Sanz OA, Martinez PR, Guarch RT, Goñi MJ, Alcazar JL. Bilateral Leydig cell tumour of the ovary: a rare cause of virilization in postmenopausal patient. Maturitas. 2007 Jun 20;57(2):214-6. doi: 10.1016/j.maturitas.2006.11.013.
38. Doyle LM, Cussen L, McDonnell T, O'Reilly MW. Clinical Utility of GnRH Analogues in Female Androgen Excess: Highlighting Diagnostic and Therapeutic Applications. JCEM Case Rep. 2023 Sep 19;1(5):luad108. doi: 10.1210/jcemcr/luad108
39. Polisseni F, Gonçalves Júnior H, Vidal VR, Macedo FL, Lins BD, Campos JD, Mattos NA. Síndrome hiperandrogênica em mulher na pós-menopausa: relato de caso [Hyperandrogenic syndrome in a postmenopausal woman: a case report]. Rev Bras Ginecol Obstet. 2011 Aug;33(8):214-20. Portuguese. doi: 10.1590/s0100-72032011000800008.
40. Braithwaite SS, Bitterman P, DeGeest K, Lebbin DR. Postmenopausal virilization, simple ovarian cyst, and hilus cell hyperplasia--is there an association? Endocr Pract. 2001 Jan-Feb;7(1):40-3. doi: 10.4158/EP.7.1.40.
41. Gheorghisan-Galateanu AA, Terzea D, Valea A, Carsote M. MENOPAUSAL ANDROGEN EXCESS - ASSOCIATED CARDIO-METABOLIC RISK: CLUES FOR OVARIAN LEYDIG CELL TUMOUR (CASE REPORT AND MINI-REVIEW OF LITERATURE). Acta Endocrinol (Buchar). 2017 Jul-Sep;13(3):356-363. doi: 10.4183/aeb.2017.356.
42. Yetkin DO, Demirsoy ET, Kadioglu P. Pure leydig cell tumour of the ovary in a post-menopausal patient with severe hyperandrogenism and erythrocytosis. Gynecol Endocrinol. 2011 Apr;27(4):237-40. doi: 10.3109/09513590.2010.490611.
43. Gücer F, Ozyilmaz F, Balkanli-Kaplan P, Mülayim N, Aydin O. Ovarian hemangioma presenting with hyperandrogenism and endometrial cancer: a case report. Gynecol Oncol. 2004 Sep;94(3):821-4. doi: 10.1016/j.ygyno.2004.06.021.
44. Marcelino M, Nobre E, Conceição J, Lopes L, Vilar H, França Martins M, Carvalho A, André S, Horta A, De Castro JJ. Um caso raro de hiperandrogenismo tumor ovárico bilateral de células de Leydig [A rare case of hyperandrogenism: bilateral Leydig cell tumor of the ovary]. Acta Med Port. 2010 Jan-Feb;23(1):113-8. Portuguese.
45. Mpatsoulis D, Nieto J J, Lonsdale R, Fisher C, Mazibrada J. Differential diagnosis of adipocytic differentiation in androgen-secreting mature ovarian teratoma with Leydig cell hyperplasia. Gynecol Oncol Rep. 2021 May 8;36:100786.
46. Di Giacinto P, Chioma L, Vancieri G, Guccione L, Cicerone E, Ulisse S, Mariani S, Autore C, Fabbri A, Gnessi L, Moretti C. Virilizing leydig-sertoli cell ovarian tumor associated with endometrioid carcinoma of the endometrium in a postmenopausal patient: case report and general considerations. Clin Med Insights Case Rep. 2012;5:149-53. doi: 10.4137/CCRep.S10555.
47. Cvijovic G, Yamashita SA, Micic D, Kendereski A, Sumarac-Dumanovic M, Zoric S, Popovic V. Low leptin level in an obese hyperandrogenic woman--potential marker for androgen-secreting tumor. Gynecol Endocrinol. 2007 Feb;23(2):112-6. doi: 10.1080/09513590701197924.
48. Ferrinho C, Silva E, Oliveira M, Sequeira Duarte J. OvarianLeydigcelltumor and postmenopausalhirsutism with signs of virilisation. BMJ Case Rep. 2021 Mar 17;14(3):e240937. doi: 10.1136/bcr-2020-240937
49. Tutzer M, Winnykamien I, Davila Guardia J, Castelo-Branco C. Hyperandrogenism in post-menopausal women: a diagnosis challenge. GynecolEndocrinol. 2014 Jan;30(1):23-5. doi: 10.3109/09513590.2013.850661.
50. Picón MJ, Lara JI, Sarasa JL, Recasens JD, Clouet R, Gonzalo MA, Rovira A. Use of a long-actinggonadotrophin-releasinghormoneanalogue in a postmenopausal woman with hyperandrogenism due to a hiluscelltumour. Eur J Endocrinol. 2000 Jun;142(6):619. doi: 10.1530/eje.0.1420619.
51. Aljenaee K, Ali S, Cheah SK, MacEneaney O, Mulligan N, Hickey N, Tun TK, Sreenan S, McDermott JH. Markedhyperandrogenicity in a 60-year-old woman. EndocrinolDiabetesMetab Case Rep. 2017 Sep 4;2017:17-0075. doi: 10.1530/EDM-17-0075
52. Mehta JM, Miller JL, Cannon AJ, Mardekian SK, Kenyon LC, Jabbour SA. Ovarianleydigcellhyperplasia: an unusual case of virilization in a postmenopausal woman. Case Rep Endocrinol. 2014;2014:762745. doi: 10.1155/2014/762745.
53. Zhou WB, Chen N, Li CJ. A rare case of pure testosterone-secreting adrenal adenoma in a postmenopausalelderly woman. BMC EndocrDisord. 2019 Jan 23;19(1):14. doi: 10.1186/s12902-019-0342-y
54. Hussain SA, Dubil EA, De Luca-Johnson JN, Johnston M. Occult symptomatic bilateral pure Leydig cell tumors in a postmenopausal woman: a case report. Gynecol Endocrinol. 2021 Jul;37(7):672-675. doi: 10.1080/09513590.2021.1934443.
55. Pérez A, Calaf J, Webb SM, Prat J, de Leiva A. Postmenopausal androgen secreting ovarian tumour: pathophysiological implications; a case report. Eur J Obstet Gynecol Reprod Biol. 1990 Apr;35(1):97-105. doi: 10.1016/0028-2243(90)90148-t.
56. Kozan P, Chalasani S, Handelsman DJ, Pike AH, Crawford BA. A Leydig cell tumor of the ovary resulting in extreme hyperandrogenism, erythrocytosis, and recurrent pulmonary embolism. J Clin Endocrinol Metab. 2014 Jan;99(1):12-7. doi: 10.1210/jc.2013-3108.
57. Moghazy D, Sharan C, Nair M, Rackauskas C, Burnette R, Diamond M, Al-Hendy O, Al-Hendy A. Sertoli-Leydig cell tumor with unique nail findings in a post-menopausal woman: a case report and literature review. J Ovarian Res. 2014 Aug 28;7:83. doi: 10.1186/s13048-014-0083-5
58. Bailey AP, Schutt AK, Carey RM, Angle JF, Modesitt SC. Hyperandrogenism of ovarian etiology: utilizing differential venous sampling for diagnosis. Obstet Gynecol. 2012 Aug;120(2 Pt 2):476-479. doi: 10.1097/AOG.0b013e31825a711c.
59. Bühler-Christen A, Tischler V, Diener PA, Brändle M. New onset alopecia and hirsutism in a postmenopausal women. Gynecol Endocrinol. 2009 May;25(5):324-7. doi: 10.1080/09513590902730788.
60. Adefris M, Fekadu E. Postmenopausal mild hirsutism and hyperandrogenemia due to granulosa cell tumor of the ovary: a case report. J Med Case Rep. 2017 Aug 30;11(1):242. doi: 10.1186/s13256-017-1411-3.
61. Shwana S, Shrikrishnapalasuriyar N, Yin W, Vij M, Kalhan A. An Occult Leydig Cell Tumour in a Postmenopausal Woman Presenting with Alopecia and Hirsutism: A Case Report. touchREV Endocrinol. 2021 Apr;17(1):75-78. doi: 10.17925/EE.2021.17.1.75. Epub 2021 Apr 28.
62. Manieri C, Di Bisceglie C, Fornengo R, Grosso T, Zumpano E, Calvo F, Berardengo E, Volante M, Papotti M. Postmenopausal virilization in a woman with gonadotropin dependent ovarian hyperthecosis. J Endocrinol Invest. 1998 Feb;21(2):128-32. doi: 10.1007/BF03350327.
63. Souto SB, Baptista PV, Barreto F, Sousa PF, Braga DC, Carvalho D. Ovarian intratumoral 21-hydroxylase deficiency in a postmenopausal hirsute woman. Arq Bras Endocrinol Metabol. 2012 Dec;56(9):672-6. Doi: 10.1590/s0004-27302012000900012.
64. Taşdemir N, Celik C, Abalı R, Aksu E, Oznur M, Yılmaz M. A rare cause of virilization; Ovarian steroid cell tumor, not otherwise specified (NOS). J Turk Ger Gynecol Assoc. 2012;13(4):275-277.
65. Moura FS, Costa-Barbosa FA, Leao SC, Nicolau SM, Kater CE, Monteagudo PT. Confounder factors masking a Leydig-cell ovarian tumor in a post-menopausal woman treated for androgen-positive receptor breast cancer. Gynecol Endocrinol. 2017;33(9):675-679. Doi:10.1080/09513590.2017.1318373
66. Mango D, Manna P, Liberati M, et al. Steroid hormones and gonadotropins in a case of ovarian endometriosis associated with virilization. Acta Obstet Gynecol Scand. 1992;71(2):153-155. doi:10.3109/00016349209007977
67. Montoya T, Guijarro G, Elvira R, Olivar J. Virilización en una mujer posmenopáusica. Consideraciones diagnósticas y terapéuticas [Virilization of a post-menopausal woman. Diagnostic and therapeutic considerations]. Endocrinol Nutr. 2009;56(8):422-427. doi:10.1016/S1575-0922(09)72713-5
68. Klotz RK, Müller-Holzner E, Fessler S, et al. Leydig-cell-tumor of the ovary that responded to GnRH-analogue administration - case report and review of the literature. Exp Clin Endocrinol Diabetes. 2010;118(5):291-297. doi:10.1055/s-0029-1225351
69. Chico A, García JL, Matías-Guiu X, et al. A gonadotrophin dependent stromal luteoma: a rare cause of post-menopausal virilization. Clin Endocrinol (Oxf). 1995;43(5):645-649. doi:10.1111/j.1365-2265.1995.tb02931.x
70. 96.Faraj G, Di Gregorio S, Misiunas A, et al. Virilizing ovarian tumor of cell tumor type not otherwise specified: a case report. Gynecol Endocrinol. 1998;12(5):347-352. doi:10.3109/09513599809012837
71. Ali FS, Stanaway SE, Zakhour HD, Spearing G, Bowen-Jones D. A case of hirsutism due to bilateral diffuse ovarian Leydig cell hyperplasia in a post-menopausal woman. Eur J Intern Med. 2003;14(7):432-433. doi:10.1016/s0953-6205(03)00141-9
72. Demir AY, Blok BB, Brinkhuis EA, Oldenburg-Ligtenberg CP. Hyperandrogenism due to ovarian Leydig cell tumour presenting with polycythaemia. BMJ Case Rep. 2022 Jul 15;15(7):e249651. doi: 10.1136/bcr-2022-249651. PMID: 35840164; PMCID: PMC9295641.
73. Higuchi A, Tsuji S, Amano T, Kasahara K, Kimura F, Murakami T. Ovarian Leydig cell tumour diagnosis in a postmenopausal woman with uterine bleeding: a case report and literature review. J Obstet Gynaecol. 2022;42(6):2519-2521. doi:10.1080/01443615.2022.2027897
74. Fux-Otta C, Szafryk de Mereshian P, López de Corominas M, Fuster M, López CR. Hyperandrogenism produced by ovarian tumors in women at different life stages. Rev Fac Cien Med Univ Nac Cordoba. 2014;71(2):122-6.
75. Sayegh RA, DeLellis R, Alroy J, Lechan R, Ball HG. Masculinizing granulosa cell tumor of the ovary in a postmenopausal woman. A case report. J Reprod Med. 1999;44(9):821-825.
76. Baweja K, Shuster S, Awad S. The Use of Ovarian Vein Sampling to Lateralize a Virilizing Leydig Cell Ovarian Tumor. AACE Clin Case Rep. 2023;9(6):182-185. Published 2023 Jul 22. doi:10.1016/j.aace.2023.07.003
77. Alali I, Haj Hassan L, Mardini G, Hijazi N, Hadid L, Kabalan Y. Diagnostic Dilemma in Two Cases of Hyperandrogenism. Case Rep Endocrinol. 2018;2018:9041018.
78. Bahloul E, Amouri M, Masmoudi A, et al. Tumeur virilisante de l'ovaire: une cause rare d'alopécie androgénétique [Virilizing ovarian tumor: a rare cause of androgenetic alopecia]. Ann Dermatol Venereol. 2015;142(4):303-304. doi:10.1016/j.annder.2015.01.026
79. Baiocchi G, Manci N, Angeletti G, Celleno R, Fratini D, Gilardi G. Pure Leydig cell tumour (hilus cell) of the ovary: a rare cause of virilization after menopause. Gynecol Obstet Invest. 1997;44(2):141-144. doi:10.1159/000291506
80. Berbegal L, Albares MP, De-Leon FJ, Negueruela G. Alopecia and hirsutism in a postmenopausal woman as the presenting complaint of ovarian hilus (Leydig) cell tumor. Actas Dermosifiliogr. 2015;106(8):676-678. doi:10.1016/j.ad.2014.12.022
81. Bogdanou D, Meyer G, Stuecker AU, Thalhammer A, Hansmann ML, Bojunga J. A rare case of an androgen-producing stromal luteoma of the ovary in a postmenopausal woman, diagnosed by means of selective venous blood sampling. Gynecol Endocrinol. 2016;32(9):704-708. doi:10.1080/09513590.2016.1183626
82. Böhm J, Röder-Weber M, Höfler H, Kolben M. Bilateral stromal Leydig cell tumour of the ovary. Case report and literature review. Pathol Res Pract. 1991;187(2-3):348-353. doi:10.1016/S0344-0338(11)80801-0
83. Braithwaite SS, Erkman-Balis B, Avila TD. Postmenopausal virilization due to ovarian stromal hyperthecosis. J Clin Endocrinol Metab. 1978;46(2):295-300. doi:10.1210/jcem-46-2-295
84. Chantler DJ, Gordon D, Millan D, Panarelli M. Use of cetrorelix in the investigation of testosterone excess in a postmenopausal woman. BMJ Case Rep. 2011;2011:bcr0120113730. Published 2011 Apr 26. doi:10.1136/bcr.01.2011.3730
85. Cohen I, Shapira M, Cuperman S, et al. Direct in-vivo detection of atypical hormonal expression of a Sertoli-Leydig cell tumour following stimulation with human chorionic gonadotrophin. Clin Endocrinol (Oxf). 1993;39(4):491-495. doi:10.1111/j.1365-2265.1993.tb02399.x
86. Correia S, Oliveira MJ, Wen X. Ovarian Hemangioma With Stromal Luteinization. Cureus. 2022;14(9):e29438. Published 2022 Sep 22. doi:10.7759/cureus.29438
87. de Lima GR, de Lima OA, Baracat EC, Vasserman J, Burnier M Jr. Virilizing Brenner tumor of the ovary: case report. Obstet Gynecol. 1989;73(5 Pt 2):895-898
88. Duun S. Bilateral virilizing hilus (Leydig) cell tumors of the ovary. Acta Obstet Gynecol Scand. 1994;73(1):76-77. doi:10.3109/00016349409013401
89. Elhadd TA, Connolly V, Cruickshank D, Kelly WF. An ovarian lipid cell tumour causing virilization and Cushing's syndrome. Clin Endocrinol (Oxf). 1996;44(6):723-725. doi:10.1046/j.1365-2265.1996.693515.x
90. Goldman JM, Kapadia LJ. Virilization in a postmenopausal woman due to ovarian stromal hyperthecosis. Postgrad Med J. 1991;67(785):304-306. doi:10.1136/pgmj.67.785.304
91. Hansen TP, Sørensen B. Sertoli-Leydig cell tumour of the ovary--a rare cause of virilization after menopause. APMIS. 1993;101(9):663-666. doi:10.1111/j.1699-0463.1993.tb00162.x
92. Hayes FJ, Sheahan K, Rajendiran S, McKenna TJ. Virilization in a postmenopausal woman as a result of hilus cell hyperplasia associated with a simple ovarian cyst. Am J Obstet Gynecol. 1997;176(3):719-720. doi:10.1016/s0002-9378(97)70580-5
93. Herrera JD, Davidson JA, Mestman JH. Hyperandrogenism due to a testosterone-secreting Sertoli-Leydig cell tumor associated with a dehydroepiandrosterone sulfate-secreting adrenal adenoma in a postmenopausal woman: case presentation and review of literature. Endocr Pract. 2009;15(2):149-152. doi:10.4158/EP.15.2.149
94. Huang RS, Covinsky M, Zhang S. Bilateral ovarian capillary hemangioma with stromal luteinization and hyperandrogenism. Ann Clin Lab Sci. 2013;43(4):457-459.
95. Jarabak J, Talerman A. Virilization due to a metastasizing granulosa cell tumor. Int J Gynecol Pathol. 1983;2(3):316-324. doi:10.1097/00004347-198303000-00009
96. Kim Y, Marjoniemi VM, Diamond T, Lim A, Davis G, Murrell D. Androgenetic alopecia in a postmenopausal woman as a result of ovarian hyperthecosis. Australas J Dermatol. 2003;44(1):62-66.
97. Kim YT, Kim SW, Yoon BS, Kim SH, Kim JH, Kim JW, Cho NH. An ovarian steroid cell tumor causing virilization and massive ascites. Yonsei Med J. 2007 Feb 28;48(1):142-6. doi: 10.3349/ymj.2007.48.1.142. PMID: 17326260; PMCID: PMC2628006.
98. Krug E, Berga SL. Postmenopausal hyperthecosis: functional dysregulation of androgenesis in climacteric ovary. Obstet Gynecol. 2002;99(5 Pt 2):893-897. doi:10.1016/s0029-7844(01)01588-5
99. Langevin TL, Maynard K, Dewan A. Bilateral microscopic Leydig cell ovarian tumors in the postmenopausal woman. BMJ Case Rep. 2020;13(12):e236427. Published 2020 Dec 22. doi:10.1136/bcr-2020-236427
100. Larrea AL, González VR, Knoblovits P, Gil SJ. Virilización y enfermedad metabólica en mujer postmenopáusica relacionado a hiperplasia e hipertecosis ovárica [Virilization and metabolic disease in postmenopausal women related to ovarian hyperplasia and hypertecosis]. Rev Fac Cien Med Univ Nac Cordoba. 2021;78(2):193-196. Published 2021 Jun 28. doi:10.31053/1853.0605.v78.n2.32136
101. Marcelino M, Nobre E, Conceição J, et al. Um caso raro de hiperandrogenismo tumor ovárico bilateral de células de Leydig [A rare case of hyperandrogenism: bilateral Leydig cell tumor of the ovary]. Acta Med Port. 2010;23(1):113-118.
102. Matuszczyk A, Petersenn S, Lahner H, et al. Androgenproduzierender Leydig-Zell-Tumor des Ovars als Ursache von Hirsutismus bei einer postmenopausalen Frau [Leydig cell tumor as a cause of hirsutism in a postmenopausal woman]. Med Klin (Munich). 2007;102(3):259-262. doi:10.1007/s00063-007-1032-5
103. Mrozińska S, Kiałka M, Doroszewska K, Milewicz T, Jach R. Hiperandrogenemia pochodzenia jajnikowego u kobiety po menopauzie z towarzyszacym gruczolakiem nadnercza--opis przypadku [The ovarian origin of hiperandrogenism in the postmenopausal woman the adrenal adenoma--a case report]. Przegl Lek. 2015;72(7):387-390.
104. Baramki TA, Leddy AL, Woodruff JD. Bilateral hilus cell tumors of the ovary. Obstet Gynecol.
105. de Lima GR, de Lima OA, Baracat EC, Vasserman J, Burnier M Jr. Virilizing Brenner tumor of the ovary: case report. Obstet Gynecol. 1989;73(5 Pt 2):895-898.
106. Higuchi A, Tsuji S, Amano T, Kasahara K, Kimura F, Murakami T. Ovarian Leydig cell tumour diagnosis in a postmenopausal woman with uterine bleeding: a case report and literature review. J Obstet Gynaecol. 2022;42(6):2519-2521. doi:10.1080/01443615.2022.2027897
107. Cserepes E, Szücs N, Patkós P, et al. Ovarian steroid cell tumor and a contralateral ovarian thecoma in a postmenopausal woman with severe hyperandrogenism. Gynecol Endocrinol. 2002;16(3):213-216.
108. Vouza E, Kairi-Vassilatou E, Kleanthis CK, Hasiakos N, Salakos N, Kondi-Pafiti A. A rare ovarian Leydig cell tumor (hilar type) causing virilization in a postmenopausal woman. Eur J Gynaecol Oncol. 2011;32(5):557-559.
109. Salman M P, Cuello F M, Kolbach M, Gejman R, Arteaga U E. Hiperandrogenismo avanzado en una mujer postmenopáusica. Caso clínico [Virilization caused by an ovarian tumor. Report of one case]. Rev Med Chil. 2011;139(8):1066-1070.
110. Roux-Guinot S, Gorin I, Vadrot D, Djid R, Bethoux JP, Escande JP. Alopécie androgénétique révélant une tumeur ovarienne androgéno-secrétante [Androgenic alopecia revealing an androgen secreting ovarian tumor]. Ann Dermatol Venereol. 2001;128(11):1241-1244.
111. Stephens JW, Katz JR, McDermott N, MacLean AB, Bouloux PM. An unusual steroid-producing ovarian tumour: case report. Hum Reprod. 2002;17(6):1468-1471. doi:10.1093/humrep/17.6.1468
112. van Heyningen C, MacFarlane IA, Diver MJ, Muronda C, Tuffnell D. Virilization due to ovarian hyperthecosis in a postmenopausal woman. Gynecol Endocrinol. 1988;2(4):331-338. doi:10.3109/09513598809107656
113. Vasiloff J, Chideckel EW, Boyd CB, Foshag LJ. Testosterone-secreting adrenal adenoma containing crystalloids characteristic of Leydig cells. Am J Med. 1985;79(6):772-776. doi:10.1016/0002-9343(85)90531-5
114. Ozgun MT, Batukan C, Turkyilmaz C, Dolanbay M, Mavili E. Selective ovarian vein sampling can be crucial to localize a Leydig cell tumor: an unusual case in a postmenopausal woman. Maturitas. 2008;61(3):278-280. doi:10.1016/j.maturitas.2008.09.003
115. Shakir MKM, Snitchler AN, Vietor NO, Mai VQ, Hoang TD. Bilateral Ovarian Leydig Cell Tumors in a Postmenopausal Woman Causing Hirsutism and Virilization. AACE Clin Case Rep. 2020;7(1):26-28. Published 2020 Dec 28. doi:10.1016/j.aace.2020.11.004
116. Silva PD, Sorensen ML, Reynertson R, Virata RL, Mahairas GH. Laparoscopic removal of virilizing hilar cell tumor in a postmenopausal patient. J Am Assoc Gynecol Laparosc. 1997;4(4):499-502. doi:10.1016/s1074-3804(05)80047-7
117. Singh P, Deleon F, Anderson R. Steroid cell ovarian neoplasm, not otherwise specified: a case report and review of the literature. Case Rep Obstet Gynecol. 2012;2012:253152. doi:10.1155/2012/253152 T.
118. Baramki TA, Leddy AL, Woodruff JD. Bilateral hilus cell tumors of the ovary. Obstet Gynecol. 1983;62(1):128-131.
119. Cserepes E, Szücs N, Patkós P, et al. Ovarian steroid cell tumor and a contralateral ovarian thecoma in a postmenopausal woman with severe hyperandrogenism. Gynecol Endocrinol. 2002;16(3):213-216.
120. Delibasi T, Erdogan MF, Serinsöz E, Kaygusuz G, Erdogan G, Sertçelik A. Ovarian hilus-cell hyperplasia and high serum testosterone in a patient with postmenopausal virilization. Endocr Pract. 2007;13(5):472-475. doi:10.4158/EP.13.5.472
121. Feiz F, Tehranian A, Heidary SS, Seifollahi A. New-Onset Type 2 Diabetes and Virilization in a Benign Sex Cord Tumor. J Coll Physicians Surg Pak. 2016;26(11):S89-S91.
122. Godlewski G, Nguyen Trong AH, Tang J, Semler-Collery R, Joujoux JM, Gaujoux AF. Virilizing adrenal ganglioneuroma containing Leydig cells. Acta Chir Belg. 1993;93(4):181-184.
123. Wu DH, McMurtrie DG, Hirsch SD, Johnston CM. Postmenopausal hyperandrogenism caused by a benign cystic teratoma: a case report. J Reprod Med. 2008 Feb;53(2):141-4.
