# Supplementary material for: Postmenopausal ovarian hyperandrogenism of surgically treated patients: a case report and scoping review with individual patient’s data analysis
Source: Front Endocrinol (Lausanne). 2025 Aug 1;16:1495930. doi: 10.3389/fendo.2025.1495930 (PMC12353694; doi:10.3389/fendo.2025.1495930)
Supplement: Supplementary file 2 [file Table2.docx]

Supplementary table 2

**Quality assessment of case series included in the systematic review according to Joanna Briggs Institute Critical Appraisal Checklist for Case Series^#^**

|  |  | **Question** | | | | | | | | | |
| --- | --- | --- | --- | --- | --- | --- | --- | --- | --- | --- | --- |
| Reference | **N** | **1** | **2** | **3** | **4** | **5** | **6** | **7** | **8** | **9** | **10** |
|  | 5 | Y | Y | Y | N | Y | Y | Y | Y | Y | Y |
|  | 2 | Y | Y | Y | N | Y | Y | Y | Y | Y | Y |
|  | 2 | Y | Y | Y | N | Y | Y | Y | Y | Y | Y |
|  | 5 | Y | Y | Y | N | Y | Y | Y | Y | Y | Y |
|  | 4 | Y | Y | Y | N | Y | Y | Y | Y | Y | Y |
|  | 2 | Y | Y | Y | N | Y | Y | Y | Y | Y | Y |
|  | 2 | Y | Y | Y | N | Y | Y | Y | Y | Y | Y |
|  | 8 | Y | Y | Y | N | Y | Y | Y | Y | Y | Y |
|  | 34 | Y | Y | Y | N | Y | Y | Y | Y | Y | Y |
|  | 24 | Y | Y | Y | N | Y | Y | Y | Y | Y | Y |
|  | 2 | Y | Y | Y | N | Y | Y | Y | Y | Y | Y |
|  | 2 | Y | Y | Y | N | Y | Y | Y | Y | Y | Y |
|  | 2 | Y | Y | Y | N | Y | Y | Y | Y | Y | Y |
|  | 2 | Y | Y | Y | N | Y | Y | Y | Y | Y | Y |
|  | 4 | Y | Y | Y | N | Y | Y | Y | Y | Y | Y |
|  | 4 | Y | Y | Y | N | Y | Y | Y | Y | Y | Y |
|  | 4 | Y | Y | Y | N | Y | Y | Y | Y | Y | Y |
|  | 2 | Y | Y | Y | N | Y | Y | Y | Y | Y | Y |
|  | 12 | Y | Y | Y | N | Y | Y | Y | Y | Y | Y |
|  | 4 | Y | Y | Y | N | Y | Y | Y | Y | Y | Y |
|  | 6 | Y | Y | Y | N | Y | Y | Y | Y | Y | Y |
|  | 4 | Y | Y | Y | N | Y | Y | Y | Y | Y | Y |
|  | 42 | Y | Y | Y | N | Y | Y | N | Y | Y | Y |
|  | 2 | Y | Y | Y | N | Y | Y | Y | Y | Y | Y |
|  | 2 | Y | Y | Y | N | Y | Y | Y | Y | Y | Y |
|  | 18 | Y | Y | Y | N | Y | Y | Y | Y | Y | Y |
|  | 2 | Y | Y | Y | N | Y | Y | Y | Y | Y | Y |
|  | 2 | Y | Y | Y | Y | Y | Y | Y | N | N | Y |

Answers

1. Were there clear criteria for inclusion in the case series?
2. Was the condition measured in a standard, reliable way for all participants included in the case series?
3. Were valid methods used for identification of the condition for all participants included in the case series?
4. Did the case series have consecutive inclusion of participants?
5. Did the case series have complete inclusion of participants?
6. Was there clear reporting of the demographics of the participants in the study?
7. Was there clear reporting of clinical information of the participants?
8. Were the outcomes or follow-up results of cases clearly reported?
9. Was there clear reporting of the presenting sites’/clinics’ demographic information?
10. Was statistical analysis appropriate?

**Yes: Y/No: N/Unclear: U/Not Applicable: N.A.**

**^#^** Moola S, Munn Z, Tufanaru C, Aromataris E, Sears K, Sfetcu R, Currie M, Lisy K, Qureshi R, Mattis P, Mu P. Chapter 7: Systematic reviews of etiology and risk. In: Aromataris E, Munn Z (Editors)*. JBI Manual for Evidence Synthesis.* JBI, 2020. Available from https://synthesismanual.jbi.global. https://doi.org/10.46658/JBIMES-20-08

**CASE SERIES REFERENCES**

1. Ajith S, Beena G, Mathew NM, Omana EK. Postmenopausal hyperandrogenism of ovarian origin: A clinicopathologic study of five cases. J Midlife Health. 2016 Oct-Dec;7(4):189-192. doi: 10.4103/0976-7800.195699.
2. Bahaeldein E, Brassill MJ. Utilisation of gonadotrophin-releasing hormone (GnRH) analogue to differentiate ovarian from adrenal hyperandrogenism in postmenopausal women. Endocrinol Diabetes Metab Case Rep. 2018;2018:18-0084. doi: 10.1530/EDM-18-0084.
3. Ashawesh K, Abdulqawi R, Redford D, Barton D. Postmenopausal hyperandrogenism of ovarian origin: diagnostic and therapeutic difficulties. Endocr J. 2007 Aug;54(4):647. doi: 10.1507/endocrj.k07-115.
4. Pelusi C, Forlani G, Zanotti L, Gambineri A, Pasquali R. No metabolic impact of surgical normalization of hyperandrogenism in postmenopausal women with ovarian androgen-secreting tumours. Clin Endocrinol (Oxf). 2013 Apr;78(4):533-8. doi: 10.1111/j.1365-2265.2012.04438.x.
5. Honoré LH, Chari R, Mueller HD, Cumming DC, Scott JZ. Postmenopausal hyperandrogenism of ovarian origin. A clinicopathologic study of four cases. Gynecol Obstet Invest. 1992;34(1):52-6. doi: 10.1159/000292725.
6. Arteaga E, Martinez A, Jaramilo J, Villaseca P, Cuello M, Valenzuela P, Gejman R, Blumel JE. Postmenopausal androgen-secreting ovarian tumors: challenging differential diagnosis in two cases. Climacteric. 2019 Aug;22(4):324-328. doi: 10.1080/13697137.2018.1549214.
7. Sehemby M, Bansal P, Sarathi V, Kolhe A, Kothari K, Jadhav-Ramteke S, Lila AR, Bandgar T, Shah NS. Virilising ovarian tumors: a single-center experience. Endocr Connect. 2018 Dec;7(12):1362-1369. doi: 10.1530/EC-18-0360.
8. Zou M, Chen R, Wang Y, He Y, Wang Y, Dong Y, Li J. Clinical and ultrasound characteristics of virilizing ovarian tumors in pre- and postmenopausal patients: a single tertiary center experience. Orphanet J Rare Dis. 2021 Oct 12;16(1):426. doi: 10.1186/s13023-021-02057-z.
9. Yance VRV, Marcondes JAM, Rocha MP, Barcellos CRG, Dantas WS, Avila AFA, Baroni RH, Carvalho FM, Hayashida SAY, Mendonca BB, Domenice S. Discriminating between virilizing ovary tumors and ovary hyperthecosis in postmenopausal women: clinical data, hormonal profiles and image studies. Eur J Endocrinol. 2017 Jul;177(1):93-102. doi: 10.1530/EJE-17-0111.
10. Rocha T, Crespo RP, Yance VVR, Hayashida SA, Baracat EC, Carvalho F, Domenice S, Mendonca BB, Gomes LG. Persistent Poor Metabolic Profile in Postmenopausal Women With Ovarian Hyperandrogenism After Testosterone Level Normalization. J Endocr Soc. 2019 Apr 4;3(5):1087-1096. doi: 10.1210/js.2018-00405
11. Klimek M, Radosz P, Lemm M, Szanecki W, Dudek A, Pokładek S, Piwowarczyk M, Poński M, Cichoń B, Kajor M, Witek A. Leydig cell ovarian tumor - clinical case description and literature review. Prz Menopauzalny. 2020 Sep;19(3):140-143. doi: 10.5114/pm.2020.99578.
12. Caulkins M, Ricciuti J, Desouki M, Mager KL. Varying Phenotypes of Leydig Cell Hyperplasia of the Ovary: Two Case Reports. Case Rep Obstet Gynecol. 2023 Aug 8;2023:7178201. doi: 10.1155/2023/7178201.
13. Hofland M, Cosyns S, De Sutter P, Bourgain C, Velkeniers B. Leydig cell hyperplasia and Leydig cell tumour in postmenopausal women: report of two cases. Gynecol Endocrinol. 2013 Mar;29(3):213-5. doi: 10.3109/09513590.2012.705375.
14. Dueñas AR, Sánchez Dueñas LE, Sánchez VT, García Rico ID. Female Androgenetic Alopecia with Male Pattern Caused by an Androgen-Producing Tumor. Int J Trichology. 2020 May-Jun;12(3):121-123. doi: 10.4103/ijt.ijt_93_20
15. Di Bisceglie C, Brocato L, Tagliabue M, Bertagna A, Gianotti L, Ghigo E, Manieri C. Acute goserelin administration inhibits gonadotropin and androgen secretion in post-menopausal women with ovarian hyperandrogenism. J Endocrinol Invest. 2003 Mar;26(3):206-10. doi: 10.1007/BF03345158.
16. Fanta M, Fischerová D, Indrielle-Kelly T, Koliba P, Zdeňková A, Burgetová A, Vrbíková J. Diagnostic pitfalls in ovarian androgen-secreting (Leydig cell) tumours: case series. J Obstet Gynaecol. 2019 Apr;39(3):359-364. doi: 10.1080/01443615.2018.1517148.
17. Castell AL, Hieronimus S, Chevallier A, Sadoul JL, Galand-Portier MB, Delotte J, Fénichel P. Hyperthécoseovarienne post-ménopausique [Post-menopausalovarianhyperthecosis]. Gynecol Obstet Fertil. 2012 May;40(5):316-9. French. doi: 10.1016/j.gyobfe.2011.07.049.
18. Sherf S, Martinez D. Leydig cell tumor in the post-menopausal woman: case report and literature review. Acta Biomed. 2016 Jan 16;87(3):310-313.
19. Shah S, Torres C, Gharaibeh N. Diagnostic Challenges in Ovarian Hyperthecosis: Clinical Presentation with Subdiagnostic Testosterone Levels. Case Rep Endocrinol. 2022 Jan 18;2022:9998807. doi: 10.1155/2022/9998807.
20. Palha A, Cortez L, Tavares AP, Agapito A. Leydig cell tumour and mature ovarian teratoma: rare androgen-secreting ovarian tumours in postmenopausal women. BMJ Case Rep. 2016 Nov 1;2016:bcr2016215985. doi: 10.1136/bcr-2016-215985
21. Vaikkakara S, Al-Ozairi E, Lim E, Advani A, Ball SG, James RA, Quinton R. The investigation and management of severe hyperandrogenism pre- and postmenopause: non-tumor disease is strongly associated with metabolic syndrome and typically responds to insulin-sensitization with metformin. Gynecol Endocrinol. 2008 Feb;24(2):87-92. Doi: 10.1080/09513590701807100.
22. Barth JH, Jenkins M, Belchetz PE. Ovarian hyperthecosis, diabetes and hirsuties in post-menopausal women. Clin Endocrinol (Oxf). 1997;46(2):123-128. Doi:10.1046/j.1365-2265.1997.1050916.x
23. Luque-Ramírez M, Nattero-Chávez L, Rodríguez-Rubio Corona C, Ortiz-Flores AE, García-Cano AM, Rosillo Coronado M, Pérez Mies B, Ruz Caracuel I, Escobar-Morreale HF. Postmenopausal onset of androgen excess: a diagnostic and therapeutic algorithm based on extensive clinical experience. J Endocrinol Invest. 2024 Feb 13. Doi: 10.1007/s40618-023-02297-9.
24. Ballesteros-Pomar MD, Vidal-Casariego A. A hidden cause of virilization in postmenopausal women. Endocrinol Nutr. 2014;61(8):436-438. doi:10.1016/j.endonu.2014.04.004
25. Kolben M, Jänicke F, Böhm J, Röder-Weber M, Graeff H, Höfler H. Androgenisierung in der Postmenopause bei seltenen Ovarialtumoren--zwei Fallberichte [Androgenization in postmenopause in rare ovarian tumors—2 case reports]. Geburtshilfe Frauenheilkd. 1990;50(8):650-652. doi:10.1055/s-2008-1026519
26. A Sarfati J, Bachelot A, Coussieu C, Meduri G, Touraine P; Study Group Hyperandrogenism in Postmenopausal Women. Impact of clinical, hormonal, radiological, and immunohistochemical studies on the diagnosis of postmenopausal hyperandrogenism. Eur J Endocrinol. 2011;165(5):779-788. Doi:10.1530/EJE-11-0542
27. Alali I, Haj Hassan L, Mardini G, Hijazi N, Hadid L, Kabalan Y. Diagnostic Dilemma in Two Cases of Hyperandrogenism. *Case Rep Endocrinol*. 2018;2018:9041018. Published 2018 Jun 27. doi:10.1155/2018/9041018
28. Garzia E, Galiano V, Guarnerio PP, Marconi AM. Diagnostic pitfalls in ovarian androgen-secreting tumors in postmenopausal women with rapidly progressed severe hyperandrogenism. Post Reprod Health. 2025 Mar;31(1):45-49. doi: 10.1177/20533691241304541.
